# Implementation of sexual and gender minority health curricula in health care professional schools: a qualitative study

**DOI:** 10.1186/s12909-020-02045-0

**Published:** 2020-05-06

**Authors:** Mandi L. Pratt-Chapman

**Affiliations:** The George Washington University, GW Cancer Center, 2600 Virginia Ave, #324, Washington, DC 20037 USA

**Keywords:** LGBTQI, Sexual and gender minority, Curricula, Implementation

## Abstract

**Background:**

Lesbian, gay, bisexual, transgender, queer, and intersex people—inclusively termed “sexual and gender minorities”—have unique health and health care needs that are not being met by most healthcare providers due to lack of training in health care professional schools. The purpose of this qualitative study was to examine implementation factors for advancing sexual and gender minority health professional student curricula in academic settings.

**Methods:**

Semi-structured interviews guided by the Consolidated Framework for Implementation Research (CFIR) were conducted with sixteen curricular champions to identify factors relevant to curricular adoption, integration, and sustainment. Themes were coded using a hybrid of deductive and inductive approaches and reported using major CFIR domains.

**Results:**

Facilitators supporting implementation of sexual and gender minority health curricula included collaboration among multiple stakeholders, alignment of formal and hidden curricula, fostering an organizational culture that valued inclusion and diversity, engagement with external subject matter experts or faculty with content expertise, and thoughtful and inclusive planning.

**Conclusion:**

This study contributes to health care professional education research as well as to implementation science. Facilitators that were identified in this study can be used to increase the adoption, integration, and sustainment of sexual and gender minority health curricula in diverse academic settings.

## Background

Lesbian, gay, bisexual, transgender, queer, and intersex people—inclusively termed “sexual and gender minorities” (SGM)—have unique health care needs not being met by most health care providers [1–3]. SGM patients have documented health disparities related to chronic disease, mental health, sexually transmitted infections, substance use, and intimate partner violence that are exacerbated by stigma, trauma, and medical mistrust [1]. Lack of provider competence—including knowledge of and attitudes toward SGM, culturally-affirming behaviors, and clinical management strategies—have a direct impact on SGM patient experiences with health care, healthcare seeking behaviors, and health outcomes.

However, health care professional student curricula focused on SGM content is minimal: Medical students receive a median of 5 h [1], nursing students a median of 2.12 h [4], and dental students an average of 3.68 h [5] of health content specific to SGM populations. Published studies of healthcare professional learning interventions to improve one or more components of clinical competence and/or to moderate student bias against SGM patients in academic healthcare settings are relatively rare. Learning interventions that focus on SGM health to improve pre-graduate student knowledge, attitudes, and behaviors regarding the unique needs of SGM patients are even fewer.

Given the relative newness of the introduction of SGM curricular content to health care professional student education, this study aimed to examine implementation factors that advanced SGM health curricular adoption, integration, and sustainment in academic settings for medical, nursing, and pharmacy students. The focus on implementation is intended to inform efforts of future SGM curricular champions in diverse settings.

## Methods

### Recruitment

A literature review was conducted to identify SGM curricular interventions published within the last 5 years. Eligible studies were clustered by institution (see Table 1). Champions for SGM curricular change from 21 institutions in the U.S. and U.K. were invited for interview via email and/or phone. Authors of curricular interventions that focused on improving health care professional competence in SGM health that were systematically evaluated and published within the past 5 years or non-author colleagues who made substantial contributions to the curricular intervention were eligible for an interview invitation. See Table 1 for institutions whose faculty, staff, student leaders or institutional collaborators were eligible for interview. The Table also includes information to critically assess the evaluation rigor of the learning intervention, (e.g., use of a comparison group, use of a validated scale, and unique study design considerations). Studies not focused on student learners, interventions that did not report evaluation results, and interventions without any evaluation component were excluded.

**Table 1:** Critical assessment of SGM curricular learning interventions for students of health professions

Institution	Included comparison group?	Use of validated scale?	Level of intervention	Inclusion and exclusion (and unique considerations)
Boonshoft School of Medicine, Wright State University [6]	No	No	Individual	Included
Boston University School of Medicine [7–10]	No [7–10]	No [7–10]	Individual	Included
Case Western University School of Medicine [11, 12]	No [11, 12]	No [11, 12]	Individual	Included
Columbia University [13, 14]	No [13, 14]	No [13, 14]	Individual	[13]Excluded: Results not reported
				[14]Included: One of few longitudinal designs; 90-day follow up showed return to baseline scores
Hunter College of the City University of New York [15]	Yes	Yes	Individual	Included
Johns Hopkins School of Medicine [16]	No	No	Individual	Included
May Medical School, Rochester, MN [17]	No	No	Individual	Included: One of few longitudinal designs; 30-day follow-up showed retention of knowledge gains posttest
Northwestern University Feinberg School of Medicine [18]	No	No	Individual	Included
San Diego State University [19]	No	No	Individual	Included
School of Nursing, San Francisco State University [20]	No	N/A	N/A	Excluded: Focus is on practitioners, not student learning
University of Bristol (United Kingdom) [21]	No	No	Individual	Included
University of California Davis School of Medicine [22]	No	N/A	Systems	Included
University of California San Diego School of Medicine [23]	No	No	Individual	Included
University of California, San Francisco [24]	No	N/A	Individual	Excluded: No formal assessment conducted
University of California, San Francisco [25–27]	No [25–27]	No [25–27]	Individual [25, 26]	Included
			Individual [27]	
University of Connecticut School of Nursing & University of Central Florida College of Nursing [28]	No	Yes	Individual	Included
University of Illinois at Chicago, School of Nursing [29]	No	No	Individual	Excluded. No formal evaluation conducted.
University of Louisville, Kentucky [30–34]	No [30]	No [30]	Individual and Systems	Included [31, 32]: Describes the innovation and outcomes of the innovation [30, 34]; Components of the larger systems-level work; [33] Formative work
	N/A [31]	N/A [31]		
	Yes [32]	Yes [32]		
	N/A [33]	N/A [33]		
	No [34]	No [34]		
University of Pennsylvania Perelman Schools of Medicine, Nursing, and Dentistry [2, 35, 36]	Yes [2]	No [2]	Individual [2]	Included
	No [35]	N/A [35]	Systems [35]	
	N/A [36]	N/A [36]	Systems [36]	
University of Pittsburg School of Medicine [37]	No	No	Individual	Included
University of Wisconsin-Oshkosh, School of Nursing [38]	No	No	Individual	Included
Vanderbilt University School of Medicine [39]	No	No	Individual	Included
Wegman’s School of Pharmacy, Rochester, New York [40]	No	No	Individual	Included
Wesleyan University [41]	No	Yes	Individual	Included
Yale University School of Medicine [42]	N/A	N/A	Systems	Excluded: Not a study

Each potential interviewee was contacted by email or phone up to three times. If an author identified a colleague as a better informant of the curricular intervention, interview with the colleague was sought in place of the author. A total of 16 faculty and student leaders agreed to be interviewed from January to February 2019. Interviews were conducted by phone using the WebEx platform, recorded via WebEx and then saved to a secure Box folder. Interviews were transcribed using Rev.com (San Francisco, CA) and coded by the author using NVivo 12 software by QSR International. No incentives were provided to interviewees.

### Instrumentation

The interview guide was structured based on constructs from the Consolidated Framework for Implementation Research (CFIR) [43]. The CFIR was created as an overarching typology to consolidate the overlapping implementation theories that arose in recent years in the attempt to expedite efficacious interventions into effective health care practice. The semi-structured interview protocol included questions about the major domains of the CFIR: these include individual characteristics, intervention characteristics, inner setting, process, and outer setting (See Supplemental Material). Individual characteristics refer to knowledge, beliefs, skills, and other characteristics of individuals who are needed to implement the intervention—in this case the intervention was curriculum specifically focused on SGM health for students in an academic setting. Intervention characteristics are aspects that might influence implementation of the intervention, such as the relative advantage or complexity of the intervention. Inner setting refers to features of the organization implementing an intervention, such as the organizational culture or leadership engagement. Process refers to the specific strategies used to implement the intervention, such as engaging stakeholders. Outer setting refers to external factors that could influence implementation of the intervention [43].

### Procedures

The following criteria were used to establish trustworthiness of qualitative findings. Credibility was established through feedback by senior faculty on research design, instrumentation, and coding approach. Transferability of findings was established through thick description and transparency of data collection protocols [44]. The author documented self-reflexive memos throughout interviewing and data analysis. Process documentation contributed to confirmability through senior faculty auditing. Member checking [44] was used by sharing thematic codes and specific quotations exemplifying themes with all interviewees via email and encouraging participant feedback on the accuracy of analysis prior to data reporting. Furthermore, a colleague with expertise in SGM curricular change in two academic settings reviewed the findings to confirm transferability.

#### Anonymity and confidentiality

Interviewees were recruited based on authorship of a published learning intervention (see Table 1). Themes and quotations do not identify the interviewee nor the institution where the intervention was implemented to maintain anonymity. However, given the small number of institutions that have led curricular change in this area, it is possible that information could be suggestive of particular institutions.

#### Data management

Recordings of WebEx videos and audio files were stored in a Box folder on secure servers. Qualitative data were transcribed by uploading to Rev.com (San Francisco, CA), a secure platform that stores and transmits files using TLS 1.2 encryption and a 128-bit AES key (Myers, 2107). Transcripts were de-identified and stored in a separate Box folder available only to the author and mentoring faculty. Transcripts were uploaded into NVivo 12 for coding and analysis.

### Data analysis

The author conducted open, single coding using a mixed deductive-inductive process, beginning with examination of CFIR domains. Participants were also asked about recommendations for the field and coded as “Recommendations for Future Work.” Child and grand-child nodes were inductively coded.

## Results

### Individual characteristics: empowered, motivated institutional champions are key

Individual characteristics that emerged were focused on the level of empowerment felt by interviewees in changing SGM curriculum in their academic setting. Empowerment refers to faculty and/or students’ self-efficacy in implementing novel education in the area of SGM health. Themes included feeling empowered or motivated versus feeling disaffected. The themes of motivation and empowerment were common across nearly all interventions – motivations varied, but institutional champions with passion for SGM health were critical for success. A few interviewees mentioned feeling disaffected, either by the hierarchy of medicine that can intimidate students when noticing non-affirming care modeled by senior faculty or from burnout after years of feeling unsupported by administrators. Those who felt empowered described efforts to lead change at various levels, including at the national level.

#### Empowerment

Some interventions were student led, and some were faculty led, but for most learning interventions both faculty and student engagement were important. One student interviewee who spearheaded SGM curriculum in an urban setting said, “Honestly, I felt very shielded from institutional barriers, because of the faculty, because of our faculty sponsors.” In other settings, faculty reported being in a position to either directly change curricula within a course or feeling empowered by administrators to enact broader curricular change. In some cases, the champion was a high-level administrator. One course director said,

I called it a content change. I said that curriculum would just be the general endocrine curriculum, but I was adding a little bit of content to what the general endocrine curriculum should now be. (*laughs*) So the point is, I didn't ask anybody and so that helped.

Another faculty had authority over the entire curriculum for the medical school:

I led the efforts to create the competencies around cultural competence that we use … Now... I'm the acting associate vice-chancellor for diversity and inclusion so I oversee the full gamut of diversity efforts.

For most successful interventions, a combination of student and faculty engagement was key. Regardless of whether students or faculty initiated curricular change, successful implementation relied on at least one strong institutional champion. One doctor, who initiated curricular change when she was a medical student said:

I was in my first year and one of my friends was in a more senior year, and she'd just done a teaching week that was focused around kind of disability and diversity and things like that. And then she said, “But it was really weird, because there was nothing on … LGBT material or the demographic at all.” And I kinda said, “Well, that's a bit stupid. Maybe we should try and do something about it.”

The student noted the importance of having a strong faculty champion to support her as well as institutional supports—such as time and lecture space--to deliver curricular content to other students.

#### Motivation

Reasons for motivation varied, but most institutional champions reported being strongly motivated. Several curricular champions noted the dearth of content in the existing curricula and wanted to remedy that gap: “I graduated in 2011 … so somewhat recently. But my clinical and pre-clinical education was: ‘HIV happens more frequently to gay men. The end.’” Others were motivated by personal experiences of discrimination or hearing of others’ experiences: “I think part of it just comes from lived experience as a gay person.” One ally said after being trained through the Safe Zone Project, an initiative that raises awareness of LGBTQ implicit bias, and hosting a transgender panel for her students, “I was just astounded. And, I thought, before that point, that I was a pretty like, liberal, cool person. Two of my best friends were gay, and I thought, ‘Oh, yeah, I know all this.’ … But, I never had the opportunity to really think about what it was like to be them, and to live in, you know, our culture.” Other interviewees were motivated by a strong belief in accessible health care for all: “[I] t doesn’t matter what your personal belief system is related to transgender health or care. If you believe that people should be transgender or don’t believe that they should be is irrelevant. Their human health is what’s relevant. So we have people that can’t access healthcare based on their provider bias.” One respondent passionately spoke about discovering the biological foundation of gender dysphoria. His epiphany came from learning about failed genital surgeries on intersex children. He used this discovery to introduce the concept of biology in gender identity to students while sensitizing future physicians to the needs of transgender patients. This interviewee studies gender identity in terms of genetic material that presents along a spectrum such that “when the baby pops out, there’s certain genitals and there’s gender identity hardwired into that brain,” and these do not always align.

Only a few interviewees mentioned feeling disaffected. A few faculty champions mentioned feeling unsure how higher-level administrators would react to the introduction of SGM content in their course. One student champion said, “It’s really hard. I’ve said stuff and it’s backfired and I’ve not said stuff and felt terrible afterwards that I didn’t say anything and … I consider myself a little bit above average with this particular topic in medicine.” While not unique to SGM issues, the hierarchy in medical education intimidated some learners to speak up. A tenured faculty who felt no support or incentive to invest in SGM curricular change reported, “I’ve kind of given up … it doesn’t seem like these types of trainings are effective … so I don’t try to guilt people out, but I just try to, make people aware of how vulnerable this population is.”

### Intervention characteristics

Interventions showed wide variations in terms of the authority of institutional champions to enact change as well as topics covered. Expertise to deliver content was a major theme that affected intervention characteristics. Sustainability was influenced by the degree to which the curriculum depended on one versus many collaborating stakeholders.

#### Wide variation

Interventions were diverse, ranging from one-off, elective interventions to complete curriculum overhaul. The level of integration also varied widely from student-led projects without sustainability to course directors adopting specific content for their course to curricular leaders requiring students to demonstrate competencies in SGM health to graduate from medical school. Faculty in a school that revised 50 h of highly-integrated curricular content emphasized the importance of delivering content at relevant, teachable moments:

If we're talking about hormonal medication when we...teach about hormonal medications, they're used to treat prostate cancer, they're used to treat breast cancer, they're used to prevent … conception, and they're used for multiple other purposes, and they're also used for gender affirming care in transgender patients. So we would just integrate to that content. When we taught the sexual history, we just integrated more affirmative, inclusive language, and kind of broadening what you ask about, and what specific questions you might ask. So we didn't have a, oh, “and once you realize your patient's gay, you need to do these sort of things.” It was more...like kind of approaching the personhood and then things would unfold a little bit more naturally. When we … teach about healthcare disparities, talking about specifically the healthcare disparities for this population, and where their roots are. We talked about psychiatric treatment, and counseling specifically, teaching that conversion therapy is contraindicated … carries with it a higher risk of suicide. We're we teaching this stuff anyway without being inclusive, affirmative, or getting us closer to these goals.

In contrast, a student who led an elective intervention voiced disappointment that “I kind of had classmates feel like this was more cosmetic, elective stuff that I was teaching, or trying to teach.” Another student said she felt the content would be taken more seriously if students were tested: “I feel like you actually have to test people on the content ‘cause mine was just sort of, it was a quiz but it wasn’t actually part of the grade. And I think people take things a little more seriously when you actually grade them.”

Content for learning interventions included basic terminology, bias training, mental health considerations, social determinants of health, and transgender care considerations. Pedagogical approaches included learner reflection, community member panels, faculty self-disclosure, and didactic lecture. Almost universally, intersex content was not discussed at all. One interviewee said, “No, we don’t talk a lot about intersex and what does that mean and um, yeah, we just don’t.” The two exceptions to not addressing intersex content were having students watch the film *Intersexion* and having a legal discussion as part of a breakout session for an elective all-day student-led forum. One respondent indicated a brief mention of the definition of intersex in her four-lecture learning intervention.

A major theme was the need to increase student exposure to SGM given broadly unexamined bias and lack of basic knowledge. One interviewee mentioned that he felt successful if students took away the basic fact that sexual orientation and gender identity are different constructs: “I set realistic, low bars for myself. So my goal for the first two lectures of the transgender health elective, [was]: sexual orientation and gender identity are different. Like that was my goal. We talked about other things as well, but sometimes students need to hear that multiple times.” To address bias, many interviewees emphasized the importance of exposure to the SGM community via patient panels, “out” faculty, or experiential clinical rotations. Several interviewees emphasized the importance of narratives from the SGM community to combat bias. One pharmacy faculty said:

It's much harder to you know … say, I do not support and I will never, and then all of a sudden somebody comes out and says, “Yeah, well, my daughter, my son, my family member … ” And now all of a sudden they've done a 180 in their personal position … I think it's … easier to hold a bias on a concept. It's harder when somebody is in front of you and they're telling you their life story and they're explaining the challenges that they face and how pharmacists either helped or hurt in their personal journey. I think it's much harder to walk out of that experience and say I wasn't touched.

Another interviewee described her experience coordinating her first transgender panel for students: “But when you see them actually engaging and talking to the students, that’s where it was like, ‘This is what students need.’ They need to understand that these are individuals. To have that really open discussion with them. And it was remarkable. It was remarkable.” Many interviewees emphasized the need to counter explicit or implicit bias and the impact a small amount of exposure—such as a community panel—could yield.

#### Content expertise

Another major theme was how the availability or lack of content expertise shaped the curriculum. Content that was included tended to be guided by existing faculty expertise or external expertise—either from within the community, such as patient narratives or LGBT nonprofit organizations—or from visiting faculty from other institutions. One interviewee said they started with “content experts that we have available.” Another interviewee described early institutional efforts to broadly train faculty through external expertise: “[W] e hosted a one-day faculty development event that brought in [experts] from their respective institutions as national leaders in LGBT care and medical education related to LGBT care to develop all of our faculty that we felt would have a role in- in adapting our education and refining our education offerings to students.” Other faculty emphasized reliance on their local LGBT community organization: “[B] ecause I was so new to this and, initially the very first time I had just done a Safe Zone training, it opened my eyes. And, I asked the Gay Alliance. I kinda followed their lead about what should be incorporated.”

Several interviewees mentioned that SGM-related health curriculum was not offered by existing faculty, because they were never trained in SGM health and did not feel they had appropriate expertise. One person said colleagues feel like, “I’m not a content expert, this isn’t something I know a lot about and I don’t know where to go to find the information so it’s just easier if I don’t teach it.” Another interviewee agreed: “Faculty may want to do it but they don’t feel comfortable doing it. They don’t feel comfortable teaching it. So part of it is, you know, the curriculum, the cultural competence folks, we need to … do our homework in terms of identifying and securing the resources, the content expertise.”

#### Sustainability

For sustainability, themes of diverse stakeholder engagement and appropriate resource supports were strong. One person mentioned the ongoing effort required for this work: “And so you always need to be reengaging and engaging new people into what you’re doing or all of that hard work is just going to go by the wayside as soon as those couple of people are gone.” Level of institutional resource support—through protected faculty and/or staff time--was noted as important. These sustainability factors are detailed further in the “Inner Setting” and “Process” sections below.

### Inner setting: institutional support bolsters success

Two intertwined themes emerged as most critical to the inner setting context, or the academic context into which the SGM curriculum was introduced. Culture was an important factor, which included organizational values, organizational readiness, and the “hidden curriculum”--or the ways in which clinical practice and faculty behaviors reinforced or contradicted what was taught in the classroom. Institutional commitment, came in the form of leadership support, financial resources, protected faculty time, and staff support.

#### Culture

Cultural values emerged as a vital factor for inner context. The reasons for supporting SGM curricula varied, but alignment with the culture of the institution was helpful. A few interviewees reported institutional cultures that valued change leadership: “You know, there was just such enthusiasm from, you know, our dean, CEO, president, all the way on down, to really think about how we could be a leader and shape the national conversation and space.” Several interviewees mentioned that their institution was mission-driven, so serving “neglected populations” aligned with the institutional mission. Several others indicated a strong culture of inclusion that supported diversity initiatives. Culture was linked to organizational readiness. For those institutions that valued leadership change and inclusivity, interviewees noted that strongly supportive leadership: “[If he hesitated or hadn’t given me the top cover to really kind of push on this and be very visible, then I don’t know if we would have moved forward.”

Culture was noted in mentions of the “hidden curriculum.” One student leader said, “I mean in general, so much of med school learning is the hidden curriculum. How you model it, what words you use to describe certain patients, I mean that extends to so many things beyond sexual [or] gender minority status. And it’s really variable depending on sites as well.” In contrast to environments where the hidden curriculum was in tension with the formal curriculum, interviewees where curriculum was aligned reported a number of synergistic activities that occurred in conjunction with or following curricular change. These included direct outreach on campus to raise awareness among diverse stakeholders, new clinical services or environmental changes to make clinics more affirming, and faculty recruitment that reinforced an SGM-affirming environment. One faculty member mentioned that in response to patient complaints, their affiliated hospital started a concierge service specifically for SGM patients. Another mentioned a mentorship program that matched incoming SGM medical students with “out” faculty. The most common change that occurred to align new SGM formal curriculum with the hidden curriculum was new or expanded opportunities for students to have relevant clinical rotations. An interviewee that spearheaded a highly integrated SGM curriculum approach said, the curriculum was reinforced by “clinical programs that have now developed in support of our sexual and gender minority patients. Which, again, wasn’t anywhere on our radar, but has been very synergistic and important in allowing the educational component to work.”

#### Institutional commitment

A theme closely related to culture was institutional commitment. Commitment came in different forms, including leadership support, money, protected faculty time, and staff support. Faculty indicated that leadership support was critical: “We’ve had good leadership from the top … that has invested … and that’s been a key ingredient.” In contrast, a disaffected faculty member that did not receive support said, “If we’re really gonna be serious about this we have to think about ways that have some kind of financial and institutional support and...I can’t think of the word, but you know [how] it can get to be kind of put into the brick and mortar a bit, you know.” The same person was frustrated by the expectation of volunteerism: “I think the biggest issue for me really is, it just seems like what institutions want is somebody at the institution to take this on and to … do it as an add-on. So I’ve heard that from a lot of people and I’ve experienced that myself.” In contrast, institutions that resourced staff support attributed success to dedicated staff: “The investment the institution made in the LGBT center, I mean, this work would not have moved forward without having someone [who] is a passionate advocate and great at getting people together.” Protected faculty time was another ingredient for success, particularly for more system-wide curricular interventions: “I got time carved out to work on this and then when I became a dean and and associate vice-chancellor, I carved out faculty time to work on curriculum.”

### Process: planning and ongoing engagement are needed

In terms of the process of introducing SGM curriculum in academic health professional settings, three major themes emerged: the importance of strategically planning, consideration of contingencies (facilitators or barriers to curriculum enhancement), and responding to opposing views.

#### Strategic planning

Strategic planning included needs assessment, broad stakeholder engagement, and intentionally planning sequence and content of curriculum to optimize learning. Needs assessments ranged from broad, survey-based assessments to research mining SGM community member perspectives to informal conversations with peers. One student curriculum champion said, “I figured there’s no point in just starting something if, with stuff that I think is important, if other people are like, ‘We already know this, but we really wanted to hear about—.’ So I just tried to ask around and to see what people knew about and what they didn’t know about.”

Engagement of stakeholders ranged from informal conversations with leadership and students to entire retreats to map out new curricula. One administrative leader who took the retreat approach said:

So what we did was we invited people from various stakeholder groups … we identified folks who were curricular gatekeepers and these were the course directors, course instructors. We had people who were LGBTQ...And they would serve as our content experts and then we had in our cultural competence committee, we had the facilitators, people who could bridge because they were familiar with the curriculum but they were also familiar with cultural competence and LGBTQ health competencies … .And then we had … students or residents that could sort of give the end-user or the consumer perspective of this process.

Other interviewees leveraged standing committees or created committees to focus on curricular change. One interviewee mentioned involving community members in their advisory committee: “about eight community members, that met monthly for about a year, and reviewed all the curriculum content and offered feedback … [T] hat it was, ya know, nothing about us, without us, kind of thing.” Overall, some mix of student, faculty, and administrator engagement were consistently noted as key to successful, sustainable curricula.

Key lessons learned included: 1) making SGM curriculum implementation the purview of more than a few people; 2) starting with what you have; and 3) showing the necessity for the content. See Table 2 for major lessons learned and illustrative quotations.

**Table 2:** Lesson Learned During SGM Curricular Implementation Process

Lesson learned	Example quotation
Do not rely on one or a few people	My bigger question would be, what happens if [name] leaves? What happens, is there somebody who is going to step into that role if she goes? And I don’t know that I see this administrator saying, “Oh I really need to find somebody to make sure that we continue to”... you need more than a couple of people to understand. Because at any given point in any academic career, you’re going to have folks who come and go.
Start with what you have	I think too would be definitely to find the champions on campus that are doing this work and to support them and then try to figure out how to align the work that [is] currently going on with the medical curriculum and build from that so that it doesn’t seem like you’re starting from scratch. But that you’re building on some foundational elements that maybe seem disparate but actually can be very helpful.
Show the necessity of the content	So I think it’s sort of partly about ensuring that the feedback you get is going to be useful in helping you … either improve what you’ve got, or just kind of demonstrate that there’s a need for it. Because if everybody at the beginning said, “We really don’t need this,” and everybody at the end said, “I’m still fine. I didn’t really learn anything new,” then okay, fine, we maybe we would have given up on it.

#### Contingencies

In terms of potential barriers or facilitators to implementing SGM curricula, the revision process was the most frequently cited contingency. Several people mentioned how impacted medical school curricula are, particularly with a condensed structure. Time constraints were mentioned in tandem with potential opportunities and challenges of curricular revision. One interviewee who had worked hard on integrating SGM curricula said, “[W]e’ve subsequently gone through some real curricular revisions... where, frankly, a lot of that content and initial work was lost, because we went from a very traditional two years of basic science followed by two years of clinical rotations to a 13-month basic science integrated curriculum followed by core rotations in year two.” In contrast, others mentioned curricular revision as an opportunity:

We were undergoing curriculum renewal anyway. So, it was a good time to take advantage of that opportunity and - to kind of focus changes into all the other changes that were happening anyway. So, I mean … that's actually part of our culture here, too … Faculty are really not used to having anything be the same from one year to the next. Like...They don't have that expectation, they don't tend to roll in with the expectation, “I'm just going to get up and use my slides from last year.” … So, it made making changes a little bit easier. Our committees are not entrenched for that reason. They're just like, “Oh, is there more change? Okay, I thought I was full of change, I guess we'll have another helping.” So, um, that part was pretty easy.

In sum, curriculum revision is a time when new content can be strategically incorporated, but content that has been recently revised may not be retained if there is not broad buy-in for the importance of the content. Organizational culture that expects ongoing change was noted as a facilitator. In contrast, another interviewee described his institutional culture as “trying to stay with the tried and true... it is very much an old school culture.” This more inert culture was noted as a barrier to curricular change.

#### Responding to opposition

A few interviewees offered insights into responding to opposing views. Two major oppositions were highlighted: time constraints and conservative pushback. In response to time constraints, one interviewee noted:

We need to do a better job of intentionality. You know, and to move away from this old model of, I just spew every piece of knowledge that I have versus, what do they really need to know, what can they look up later, what's available in the database? You know, what's going to give them the foundation to be successful and I see that an awful lot in kind of, our curriculum. That it's too jam-packed and the students are too stressed to even think about adding something else in. But if we were more, if we removed redundancies and were more intentional there would be space for things that are important and quite frankly, I feel like a curriculum should be a living organism.

In terms of conservative pushback, another interviewee said,

Especially because we're down here in the Bible belt … there's some very strong opinions … There's 110,000 transgender people that live in [city] alone, you know, we're third in the country. Just giving them the facts of the data, as to the importance of what we're doing. It's human health. It has nothing to do with gender bias, has nothing to do with sexual orientation. It's related to human health. And if we all believe that all humans should have access to health, then we should all believe that this population should be able to feel comfortable getting the care they need.

In sum, institutions that integrated curricula at various levels emphasized the importance of assessing learner needs, engaging a variety of stakeholders, and responding to negative pushback. Those who integrated more systems-level curricular change also emphasized the importance of leadership and thoughtful planning to layer and diversify SGM learning opportunities over the four-year medical school experience.

### Outer setting: the larger context

Outer setting was the CFIR domain that seemed least important to successful implementation of SGM curriculum change. The broader context outside of the academic setting shaped curricular content and conversations in class, but were reported as less important for actual implementation than other factors.

#### External guidance

External guidance from credible sources and socio-political context were noted as having some effect on curriculum. For external credible resources, some interviewees that were early champions in the 2000s and early 2010s indicated lack of any real guidance from health care professional organizations, guideline bodies, or the research literature. The most cited guidance, by far, was the AAMC’s 2014 report, *Implementing Curricular and Institutional Climate Changes to Improve Health Care for Individuals who are LGBT, Gender Nonconforming, or Born with DSD: A resource for medical educators* [45]. Faculty said, “those are really our marching orders. I mean, we work from those competencies, that’s how we diagrammed out a whole curriculum. What would go where, what the sub-competencies or learning objectives would be, what the assessments would be.” Another faculty member mentioned institutional pride in achieving and maintaining leadership status in The Human Rights Campaign Healthcare Equality Index. Several interviewees mentioned how useful the *Fenway Guide to Lesbian, Gay, Bisexual and Transgender Health* [46] or Fenway Institute online resources were. Lambda Legal’s 2010 report *When Health Care Isn’t Caring* [47] was described as “groundbreaking because it was one of the few studies that really looked at LGBTQ health disparities in a comprehensive way.” The Institute of Medicine 2011 report *The Health of Lesbian, Gay, Bisexual, and Transgender People: Building a Foundation for Better Understanding* [48] was described as a “game changer.”

#### National leadership

A few curricular champions worked to effect change at a national or international level beyond their institution. One faculty member used his position of authority to influence the standard of care for intersex patients by serving on a World Professional Association for Transgender Health (WPATH) committee: “My goal, we’ll see if I succeed, my goal and this is at WPATH--where you think you’d have a friendly crowd--forget the pediatric crowd, my goal is to be able to have them say out loud that ‘when feasible,’ just put it at that because they don’t have any data so they can’t say in this situation that situation or the other situation, just simply ‘when feasible’ [genital] surgery [on intersex infants] should be delayed.” It is important to note that WPATH guidelines are for transgender patients, not intersex patients; however, there is an intersex working group in WPATH specific to transgender intersex people. This faculty leader leveraged his reputation as a transgender health expert to effect change for intersex patient standards of care within WPATH. Other faculty mentioned serving on national committees to effect change. Initiative from these leaders elevates awareness for the need for SGM health content in health care professional education at large.

#### Socio-political context

Considering sociopolitical context, a major barrier cited was the history of medicine, which has historically pathologized homosexuality:

[L] ooking at actually we as physicians, how are we contributing to that problem? And so do we have offices that are LGBT friendly? Is our staff being appropriate? And then looking at, how medicine used to say that, you know, being gay, lesbian, bisexual was a mental disorder. We didn't really help the problem. Fortunately, that's gone away, but there are still some physicians and staff who think that people like myself are an anomaly or like we need to be handled or whatever. Or they don't wanna see us. Those kind of things.

Notably, intersex status is still pathologized. A faculty interviewee noted:

We allow parents to do pretty nasty stuff to their kids if you think about it. They can brainwash them in all sorts of kooky ways (*laughs*) and then you're just dependent on your parents’ bad choices, independent of anything, even if they're totally unreasonable. So the point is kids are pretty vulnerable and we as a society, that's a value that we have, that parents are high priority.

Other examples of regional and national events were cited as socio-political facilitators or barriers to the culture change needed to create more SGM-affirming and prepared clinicians. At the state-level, faculty mentioned the real risks associated with disaffirming legal policies:

You know, like for example the [state] tried to pass a bathroom bill law in the last legislative session in 2017, and which thankfully did not go through. But, as a part of that, locally one of our equality organizations here in the state saw a significant spike in their … suicide crisis hotline from young people as the discussion on that bill was going through.

Another faculty reported that although her institution felt ready for curricular change, backlash from the religious community was not planned for: “So I think saying you’re ready and … then realizing, oh wow, the community is pulsing on this.” In contrast, greater community awareness at a national level was noted as a facilitator for dialogue among students and faculty:

There's a lot more recognition and awareness … whether it's Caitlyn Jenner or the transgender ban in the military, things that have been very, very visible … have shaped … the national dialogue … [W] e obviously live in a society that continues to evolve.

This comment references the retired Olympic gold medalist decathlete, William Bruce Jenner who changed her name to Caitlyn when she transitioned. Caitlyn Jenner and other transgender socialites have raised visibility and knowledge about transgender issues in recent years. Finally, at an administrative level, changes to insurance were noted as a positive change:

[T] he changes in insurance as well has been helpful … gender reassignment, surgery [and] hormone treatment is [*sic*] covered by insurance … [I] t gives us an impetus that, you know, if insurances are covering this and we want to be the best, um, in patient care, then we need to know about this.

Insurance reimbursement was also noted as potential leverage for obtaining higher-level buy-in among administrative leaders given emerging market opportunities.

### Recommendations for future work

In addition to examining implementation constructs, insights on where the field of SGM health education and research should go next were sought to help inform future efforts. Two major themes emerged for future directions: inadequate evaluation tools and the need to incentivize inclusion of SGM curricula. Strategies for inclusion of curricula were also offered.

#### Need for evaluation

The strongest theme that emerged when asked about what the field needs going forward was more robust evaluation tools and approaches. Universally, interviewees were unhappy with existing evaluation options in the literature, and many interviewees had plans to improve evaluation within their own settings. Faculty said, “We’ve … had some … conversations about whether or not it’d be possible to add in an OSCE, which is ask the skills that people have learned, or at least a component of it.” Another person noted the need for “having a tool of assessing how good the teaching is” and to see if people actually retain it for longer than half an hour after they go out of the session. Another faculty said:

So, we’ve done the content integration, which is great … we think that our students are learning, but we don't have any milestones in this space that are baked into our assessment tools that allow us to really know and have confidence that our students are graduating with these competencies. And, so that’s something that, you know, is on our radar, but we just haven't gotten there.

Other interviewees noted that better evaluation tools were not only needed to assess student skills but also for research.

#### Incentivizing SGM health in curricula

Several interviewees noted the importance of incentivizing inclusion of SGM content in curricula. Diverse approaches were suggested. One administrator suggested embedding SGM competence into graduation competencies: “Once you embed it in the graduation competencies, the licensing body that we call … the Liaison Committee on Medical Education, they accredit medical schools and they hold you to achieving your graduation competencies.” Another suggestion was to leverage the medical school graduation survey: “So every medical student who’s graduating is asked to take a survey of their medical school experience... It has a question on gender bias … mistreatment associated with your gender or sexual orientation. It has questions on your comfort level treating people from culturally diverse backgrounds … If your school doesn’t do well in that, then, that’s an indication that, you know... you need to fix it because if you don’t, you’re going to be in trouble by the accrediting body.” Another faculty suggested leveraging the market competition: “If you can say, ‘Oh, this institution down the street has this awesome curriculum, but we don’t. That’s a problem.’” Finally, developing off-the-shelf resources was noted as a need for the field: “We need to create things that are off the shelf. I want to talk about transgender healthcare. Okay. Do you want to talk about just LGBTQ basics? Perfect. Here’s the module. Do you want to talk about hormone replacement therapy? Great. Here’s the module. Do you want to talk about in LGBTQ patients, substance abuse and depression? Here’s where you can go or here are the resources.”

Strategies for curricular inclusion included exploration of shared values, strategic alignment of curricula and intentional exposure of students to SGM people (see Table 3).

**Table 3:** Strategies for Curricular Inclusion

Strategy	Quotation
Explore shared values to improve SGM health care	[A] ll medical schools are required to train people, to treat diverse communities. Okay. That’s an overarching value. (Faculty, Medical School)
Optimize student exposure to SGM health content by strategically aligning and layering learning at teachable moments	The big lesson is it’s very easy to implement … We’re layering it into an environment where we’re already teaching many of those things. So to layer in the idea that there is gender identity development is just not gonna be very time consuming. This isn’t like a one-week unit on all LGBT issues--and I would even be more extreme: Don’t do that. I mean you can do that also and you can certainly do cultural competence and learn terminology and things like that, but in a way this works better ...sitting next to other things that are similar to it. (Faculty, Medical School)
Offer students experiential training with SGM patients	And then the other barrier is coming up with experiential opportunities because that’s the thing that really seals it is when people have an opportunity to take care of real people, well medical trainees have a chance to take care of real people. (Faculty, Medical School)
Build partnerships with SGM community experts	[T]here’s still so much more we need to teach and so much more we need to learn and that we have to bring in our community experts to work with us (Faculty, Medical School)

## Discussion

Findings from this study support past research that has demonstrated the importance of collaboration in curricular change through stakeholder engagement [42] and the impact of aligning formal and hidden curricula [49–52]. Collaboration was reported to bolster curricular integration and sustainability of SGM curricula. Institutions that had organizational cultures that valued inclusion and diversity as well as institutional support were more likely to have leaders that provided resources to SGM curricular champions and more likely to build synergistic initiatives to further align the curriculum to support SGM-affirming care.

Content expertise emerged as critical to what and how SGM content was covered. Content expertise was addressed in a variety of ways—either starting with faculty who felt like experts, building faculty capacity through guidance from external experts, or leveraging expertise from community organizations. Content expertise as a key ingredient to SGM curricular success reinforces results of prior studies [53]. Additional contributions from this study include the importance of thoughtful planning to build faculty competence in a new topic area to make curricula less vulnerable if one person leaves.

Overall, this study provides support for implementation theory in the context of health care professional pre-graduate education SGM curricular change. In alignment with the “Recipe for successful change” offered by Ambrose the following essential ingredients proved critical for organizational change: vision, skills, incentive, resources, and an action plan [54]. Vision from institutional champions to lead change efforts emerged as foundational for SGM curricular change. Content expertise (skills) needed to be identified or developed. In Ambrose’s model, lack of incentives leads to gradual change: this theoretical assumption aligns with the recommendation from interviewees that incentives are needed to expedite SGM curricular change going forward. Resources included institutional support, usually in the form of protected faculty or staff time. Finally, needs assessment and strategic planning align with Ambrose’s call for an action plan for change. Support for Ambrose’s theory for organizational change provides important data for future researchers who wish to implement systems-level, organizational changes.

### Limitations and strengths

The major limitation of this study was singular coding of the data, resulting from institutional requirements for an independent project as part of the author’s dissertation. In addition, qualitative findings are inherently subjective. Interviewees occupy a subjective position and are likely to be influenced by social desirability bias. Examination of both facilitators and barriers and assurance of anonymity intended to minimize these risks. A strength of this study is its use of implementation theory. This is the first known study to systematically examine contextual factors associated with SGM curricular implementation in pre-graduate health care professional academic settings.

## Conclusion

Few institutions have begun to incorporate curricular change to address the gap in medical, nursing, and pharmacy student learning about SGM health. Findings from this study provide unique insights and clear strategies to aid in the adoption, integration, and sustainment of SGM health curricula into diverse academic settings.

## Supplementary information

**Additional file 1.** Interview Guide

## Data Availability

Blinded transcripts of interviews can be provided by contacting the corresponding author.

## Abbreviations

CFIR: Consolidated Framework for Implementation Science
GW: The George Washington University
LGBTQI: Lesbian, gay, bisexual, transgender, queer and intersex
SGM: Sexual and gender minority/minorities
