# Supplementary material for: Implementation of sexual and gender minority health curricula in health care professional schools: a qualitative study
Source: BMC Med Educ. 2020 May 6;20:138. doi: 10.1186/s12909-020-02045-0 (PMC7201690; doi:10.1186/s12909-020-02045-0)
Supplement: Supplementary file 1 — Additional file 1. Interview Guide [file 12909_2020_2045_MOESM1_ESM.docx]

Supplemental Material: Interview Guide

1. How did you begin your work in addressing sexual and gender minority health at your institution?
   1. Did any outside recommendations influence you in addressing sexual and gender minority health at your institution?
   2. Did student interest play any part in addressing sexual and gender minority health gaps at your institution?
2. Can you speak to specifics as to how you decided on the content of your learning intervention?
   1. How did the learning intervention you implemented reflect your initial vision?
   2. How did the learning intervention change from what you originally envisioned?
3. What lessons did you learn through implementing your sexual and gender minority health curriculum?
4. Since you wrote your paper in *[****journal/date of publication****]*, tell me what has happened with the program/ learning intervention?
   1. What contributed to its
      - 1. Success?
        2. Expansion?
        3. Shutting down?
5. Can you speak about any external factors, such as policies, incentives or competition that influenced your curricular implementation?
   1. Can you tell me about how these factors specifically influenced your project?
6. Can you tell me about any internal factors that influenced your curriculum implementation?
   1. Can you tell me about how these factors specifically influenced your project?
7. Tell me about the culture at your institution – how does that influence your work?
   1. Was there anything about the culture of the university at the time you implemented your sexual and gender minority health curriculum that influenced your project implementation?
   2. Has the culture changed since you introduced this new content to the curriculum?
8. Do you feel like your organization was ready for incorporating sexual and gender minority health content for health professional students at the time of your learning intervention? Has the readiness changed since you introduced this new content?
9. Is there anything you would like to share that we haven’t talked about?

[CLOSING]

Just a reminder that I will be writing up what we discussed today. I'll replace your name with a number so you are not identified personally.

Do I have permission to contact you after this interview?
I would like to share what I write up with you, so you can check and make sure that everything I write up makes sense and was what you meant to convey. Feel free to reach out if you have questions in the meantime.

Do I have permission to contact you if I have other questions?
